# Dose-dependent dissociation between intestinal protection and pancreatic outcome following licochalcone A treatment in severe acute pancreatitis

**DOI:** 10.3389/fphar.2026.1810680

**Published:** 2026-03-26

**Authors:** Kedong Xu, Yimin Han, Fan Chen, Yaochun Wang, Yi Miao, Zhenhua Ma, Fang Cao

**Affiliations:** 1 Department of Hepatobiliary Surgery, The First Affiliated Hospital of Xi’an Jiaotong University, Xi’an, Shaanxi, China; 2 Pancreatic Diseases Center of Xi’an Jiaotong University, Xi’an, Shaanxi, China; 3 Center for Translational Medicine, The First Affiliated Hospital of Xi’an Jiaotong University, Xi’an, Shaanxi, China

**Keywords:** dose-dependent response, gut–pancreas axis, intestinal barrier, licochalcone A, pancreatic injury, phenotypic pharmacology, severe acute pancreatitis (SAP)

## Abstract

**Introduction:**

Severe acute pancreatitis (SAP) is frequently accompanied by intestinal barrier dysfunction, systemic inflammation, and gut microbiota dysbiosis. Licochalcone A (LicA) exhibits anti-inflammatory and barrier-protective properties, but its dose-dependent effects on the gut–pancreas axis in SAP remain unclear.

**Methods:**

SAP was induced in mice by caerulein plus lipopolysaccharide. Mice were pretreated with low- or high-dose LicA prior to SAP induction. Pancreatic injury, inflammatory cell infiltration, tissue edema, and systemic and local cytokine levels were assessed by histology, immunohistochemistry, and ELISA. Ileal injury and barrier integrity were evaluated by histology and immunofluorescence. The composition of the gut microbiota was explored using 16S rRNA gene sequencing.

**Results:**

Low-dose LicA attenuated SAP severity, evidenced by lowered serum amylase and lipase levels, reduced systemic and pancreatic IL-1β and TNF-α levels, decreased inflammatory cell infiltration, and improved pancreatic histopathology. In contrast, high-dose LicA did not alleviate pancreatic injury and was associated with exacerbated acinar damage and persistent systemic inflammation. Notably, both LicA doses preserved ileal morphology, maintained tight junction protein expression, and reduced local intestinal inflammation. In an exploratory gut microbiota analysis (n = 3 per group), low-dose LicA was associated with partial normalization of SAP-associated microbial changes, whereas high-dose LicA was associated with a different pattern; these findings should be interpreted cautiously and require validation in larger cohorts.

**Conclusion:**

These findings indicate a dose-dependent dissociation between intestinal protection and pancreatic outcome following LicA treatment in SAP. While LicA preserves intestinal barrier integrity across doses, only low-dose treatment couples these local benefits to systemic and pancreatic protection, underscoring the importance of dose optimization when targeting the gut–pancreas axis. Microbiota findings are preliminary and require validation in larger cohorts.

## 1 Introduction

Acute pancreatitis is an acute inflammatory condition with a steadily increasing global incidence and represents a significant clinical challenge. Although most patients experience a mild and self-limited disease course, a clinically important subset—approximately 15–20%—develops severe acute pancreatitis (SAP). This form is characterized by extensive pancreatic tissue injury accompanied by an uncontrolled systemic inflammatory response, frequently progressing to persistent multiple organ dysfunction and substantial mortality (Boxhoorn et al., 2020). Current therapeutic strategies for SAP are largely limited to supportive care, as no pharmacological agents have been proven effective in interrupting the propagation of systemic inflammation or in protecting extra-pancreatic organs. This persistent lack of disease-modifying therapies highlights a critical unmet need to identify novel interventions that can modulate the pathological processes linking local pancreatic injury to systemic inflammatory escalation, including immune dysregulation and epithelial barrier dysfunction.

Recent findings highlight the crucial role of gut–pancreas interactions in the pathological progression of SAP. The disruption of intestinal homeostasis—often termed “gut leakage”—facilitates bacterial translocation and endotoxemia, acting as a “second hit” that amplifies systemic inflammation. While traditional views focused heavily on the physical disruption of epithelial tight junctions, more recent work highlights the importance of gut microbiota composition in modulating disease severity. SAP is frequently associated with severe dysbiosis, characterized by a reduction in beneficial commensals (e.g., *Bacteroidales*, *Clostridiales*) and an overgrowth of opportunistic Gram-negative pathogens, such as *Enterobacteriaceae* and *Enterococcus spp.* (Koh et al., 2012; Wu et al., 2023; Liu J. et al., 2024). Crucially, the expansion of these pathogenic taxa can increase the burden of lipopolysaccharide (LPS), potentially driving systemic inflammation even when the physical mucosal structure is partially preserved (Fritz et al., 2010; Zhu et al., 2019). Consequently, therapeutic strategies capable of not only restoring epithelial integrity but also correcting dysbiosis are essential for effective SAP management.

Licochalcone A (LicA), a chalcone flavonoid derived from Glycyrrhiza inflata root, is known for its anti-inflammatory, antioxidant, and epithelial integrity maintenance properties (Li et al., 2022; Liu M. et al., 2024). LicA has been shown to modulate critical signaling hubs such as NF-κB, MAPK, and PI3K/AKT, demonstrating efficacy in various inflammatory and metabolic disorders (Bhatia et al., 2023; Liu M. et al., 2024). Recent studies suggest that LicA may act as a microecological modulator by regulating intestinal microbiota composition to alleviate colitis (Zhang et al., 2021). However, pharmacological agents, particularly natural compounds, often display hormetic or biphasic dose-responses, where therapeutic outcomes may vary significantly depending on the dosage (Mattson, 2008; Jodynis-Liebert and Kujawska, 2020). Despite its broad bioactivity, the therapeutic window of LicA for managing SAP and its specific regulatory effects on the gut–pancreas axis remain unexplored. Specifically, it is unknown whether LicA intervention can synchronously mitigate intestinal barrier injury and reverse SAP-associated microbiota dysbiosis.

This study seeks to address the mechanistic gap by assessing the dose-dependent effects of LicA in a SAP mouse model induced by caerulein and lipopolysaccharide (LPS). We combined *in vivo* assessments of pancreatic injury, systemic inflammation, and intestinal barrier integrity (specifically tight junction expression) with 16S rRNA sequencing to profile gut microbiota alterations. This comprehensive method enabled the examination of the intricate relationships among LicA dosage, mucosal barrier maintenance, and microbiota modulation, offering new understanding of LicA’s safety and effectiveness in SAP treatment.

## 2 Materials and methods

### 2.1 Animals

Male C57BL/6 mice, aged 6–8 weeks and weighing 20–24 g, were obtained from the Laboratory Animal Center at Xi’an Jiaotong University, China. Animals were maintained in specific pathogen-free (SPF) conditions at 22 °C ± 2 °C with a 12-h light/dark cycle and unlimited access to food and water.

### 2.2 Ethics statement

Animal experiments complied with the Biomedical Ethics Committee of Health Science Center of Xi’an Jiaotong University (No. XJTUAE2024-2145).

### 2.3 Experimental design and animal procedures

Mice were randomly divided into six groups (n = 6 per group): NC-saline (wildtype controls with saline), NC-LicA-low (wildtype mice with low-dose LicA), NC-LicA-high (wildtype mice with high-dose LicA), SAP-saline (SAP model mice with saline), SAP-LicA-low (SAP model mice with low-dose LicA), and SAP-LicA-high (SAP model mice with high-dose LicA).

LicA dosages were determined from prior research, with 7.5 mg/kg as the low dose and 15 mg/kg as the high dose. These doses were selected to span a range previously shown to be anti-inflammatory in murine models without inducing systemic toxicity (Guo et al., 2019; Zhang et al., 2021). Mice in the LicA treatment groups received LicA via intragastric administration on alternate days for 1 week prior to model induction. The SAP model was induced using a caerulein–lipopolysaccharide protocol adapted from previously published studies (Mei et al., 2021; Liu M. et al., 2024). Mice in the SAP groups received ten hourly intraperitoneal injections of caerulein (50 μg/kg), followed by one intraperitoneal injection of lipopolysaccharide (LPS, 10 mg/kg) after the last caerulein dose. Mice were anesthetized with pentobarbital sodium (45 mg/kg) and euthanized 24 h following the initial caerulein injection. Blood samples, pancreatic tissue, and distal ileum segments were subsequently harvested for downstream analyses.

### 2.4 Reagents and antibodies

Licochalcone A (LicA) (Cat: HY-N0372), caerulein (Cat: HY-A0190) and 4′,6-diamidino-2-phenylindole (DAPI) (Cat: HY-D2868) were purchased from MedChemExpress (United States). LPS (Lipopolysaccharide, *Escherichia coli* 055 B5) (Cat: L2880) were sourced from Sigma-Aldrich (United States). Proteintech Group (China) provided ELISA kits for IL-1β (Cat: KE10003) and TNF-α (Cat: KE10002), along with primary antibodies for F4/80 (Cat: 28463-1-AP), myeloperoxidase (MPO) (Cat: 22225-1-AP), ZO-1(Cat: 21773-1-AP), and Claudin-1 (Cat: 13050-1-AP). Secondary antibodies conjugated with peroxidase and diaminobenzidine (DAB) detection kits (Cat: PV-9000) were procured from ZSGB-Bio in China, while Alexa Fluor Cy3–conjugated secondary antibodies (Cat: EK022) were acquired from Zhuangzhi Bio in China. Serum lipase (Cat: A054-1-1), malondialdehyde (MDA) (Cat: A003-1-2) and superoxide dismutase (SOD) (Cat: A001-1-2) assay kits were obtained from Nanjing Jiancheng Bio in China. Amylase assay kits (Cat: C016-1-1) were sourced from Leagene Bio located in China.

### 2.5 Assessment of pancreatic edema

Fresh pancreatic tissues were weighed (wet weight, WW) and subsequently desiccated at 60 °C for 24 h to obtain the dry weight (DW). The wet-to-dry (W/D) ratio was calculated to quantify pancreatic edema.

### 2.6 Serum enzymology

Serum amylase and lipase activities were quantified using commercial assay kits according to the manufacturers' protocols.

### 2.7 Enzyme-linked immunosorbent assay (ELISA)

Sandwich ELISA kits were used to measure IL-1β and TNF-α levels in serum, pancreatic, and ileal tissues, adhering strictly to the manufacturers' protocols.

### 2.8 Pancreatic oxidative stress assays

Pancreatic oxidative stress was assessed by measuring MDA content and SOD activity using commercial kits, and values were normalized to total protein concentration.

### 2.9 Histopathological analysis

Pancreas and ileum tissues were fixed in 4% paraformaldehyde, dehydrated using graded ethanol, embedded in paraffin, and sectioned to a thickness of 4 µm. Sections underwent hematoxylin and eosin (H&E) staining. Two independent researchers conducted histopathological scoring in a blinded manner, following established criteria (Chiu et al., 1970; Schmidt et al., 1992).

### 2.10 Immunohistochemistry (IHC) and immunofluorescence (IF)

Immunohistochemical analysis involved deparaffinizing paraffin-embedded tissue sections using xylene, followed by rehydration through a graded ethanol series. Antigen retrieval was performed using microwave heating in 0.01 M citrate buffer at pH 6.0. After blocking non-specific binding with 5% bovine serum albumin (BSA) at 37 °C, sections were incubated overnight at 4 °C with primary antibodies targeting MPO and F4/80. The following day, sections were treated with horseradish peroxidase–conjugated secondary antibodies and visualized using diaminobenzidine (DAB) as the chromogen. Whole-slide images were obtained using a Leica MP SCN400 slide scanner, and immunoreactivity was quantified as relative integral optical density (IOD) with Image-Pro Plus 6.0 software.

For immunofluorescence staining, sections were incubated overnight at 4 °C with primary antibodies against ZO-1 and Claudin-1. After thorough washing with phosphate-buffered saline, Alexa Fluor Cy3–conjugated secondary antibodies were applied. Nuclei were counterstained with DAPI. Fluorescence images were captured using a Leica TCS SP5 confocal laser scanning microscope.

### 2.11 Gut microbiota analysis

Fecal samples (n = 3 per group) were collected under sterile conditions, snap-frozen in liquid nitrogen, and stored at −80 °C. Genomic DNA was extracted, and the V3-V4 region of the 16S rRNA gene was amplified using barcode-specific primers (341F: CCTACGGGNGGCWGCAG; 806R: GGACTACHVGGGTATCTAAT). Sequencing libraries were generated and sequenced on the Illumina NovaSeq 6000 platform (Gene Denovo Biotechnology Co. Ltd, Guangzhou, China). Raw reads were deposited in the NCBI Sequence Read Archive (Accession: PRJNA938304). Bioinformatics analysis was performed using the Omicsmart platform. Alpha diversity indices were compared using Welch’s t-test, while beta diversity was assessed via the Adonis test. LEfSe (Linear discriminant analysis Effect Size) and RDA (Redundancy Analysis) were conducted to identify differentially abundant taxa and their environmental associations. Given the limited sample size (n = 3 per group), microbiome analyses, including diversity comparisons (Welch’s t-test), community-level testing (Adonis), and biomarker discovery (LEfSe), were conducted and interpreted in an exploratory manner; therefore, taxon-level and predicted functional results were regarded as hypothesis-generating and reported with appropriate caution.

### 2.12 Statistical analysis

GraphPad Prism 9.0 (GraphPad Software, San Diego, CA, United States) was used for statistical analyses. Quantitative data are expressed as mean ± SEM. Group differences were analyzed using two-way ANOVA, followed by Šidák’s multiple-comparisons test to evaluate differences between model induction conditions. A two-sided P value of less than 0.05 was considered indicative of statistical significance.

## 3 Results

### 3.1 Systemic enzymatic and inflammatory responses to licochalcone A in SAP

**FIGURE 1:**
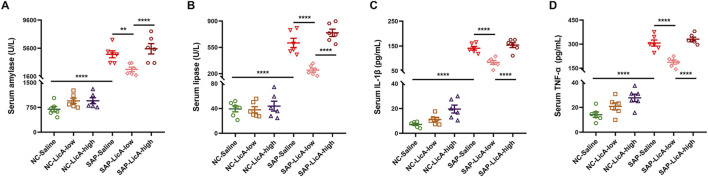
Dose-dependent effects of licochalcone A on systemic pancreatic enzymes and inflammatory cytokines in SAP. Severe acute pancreatitis (SAP) is induced by caerulein plus LPS, and serum IL-1β and TNF-α are assessed 24 h after induction. **(A,B)** Serum amylase and lipase levels. SAP markedly increases circulating enzyme levels compared with controls. Low-dose LicA reduces enzyme activities, whereas high-dose LicA does not attenuate enzyme elevation. **(C,D)** Serum IL-1β and TNF-α concentrations. SAP elevates systemic IL-1β and TNF-α levels; low-dose LicA suppresses both cytokines, while high-dose LicA shows no improvement. Data are presented as mean ± SEM (n = 6 per group). Statistical significance is indicated as *p < 0.05, **p < 0.01, ***p < 0.001, ****p < 0.0001.

Serum amylase and lipase levels were assessed 24 h post-SAP induction to determine LicA’s impact on pancreatic injury and systemic inflammation. SAP induction significantly elevated circulating amylase and lipase levels compared to controls, confirming the successful establishment of pancreatic injury (Figures 1A,B). LicA pretreatment modulated serum pancreatic enzyme levels, with differential effects observed between dosage groups: mice receiving low-dose LicA showed lower circulating amylase and lipase activities, whereas the high-dose LicA group showed persistently elevated enzyme levels.

As shown in Figures 1A,B, SAP induction resulted in marked elevations of circulating amylase and lipase levels in contrast to the control group, indicating successful establishment of pancreatic injury. LicA pretreatment differentially modulated serum pancreatic enzyme levels: the low-dose LicA group exhibited reduced circulating amylase and lipase activities relative to the SAP group, whereas the high-dose LicA group maintained persistently elevated enzyme levels.

Simultaneously, systemic inflammation was evaluated by quantifying serum levels of IL-1β and TNF-α. Serum levels of IL-1β and TNF-α were elevated in SAP mice (Figures 1C,D). LicA pretreatment influenced cytokine levels differently; the low-dose LicA group showed reduced IL-1β and TNF-α concentrations, whereas the high-dose LicA group had elevated levels similar to or higher than those in the SAP group.

These findings suggest that LicA modulates serum pancreatic enzyme release and systemic inflammatory mediator levels in SAP mice in a dose-dependent manner.

### 3.2 Pancreatic histopathology and inflammatory infiltration across licochalcone A doses

**FIGURE 2:**
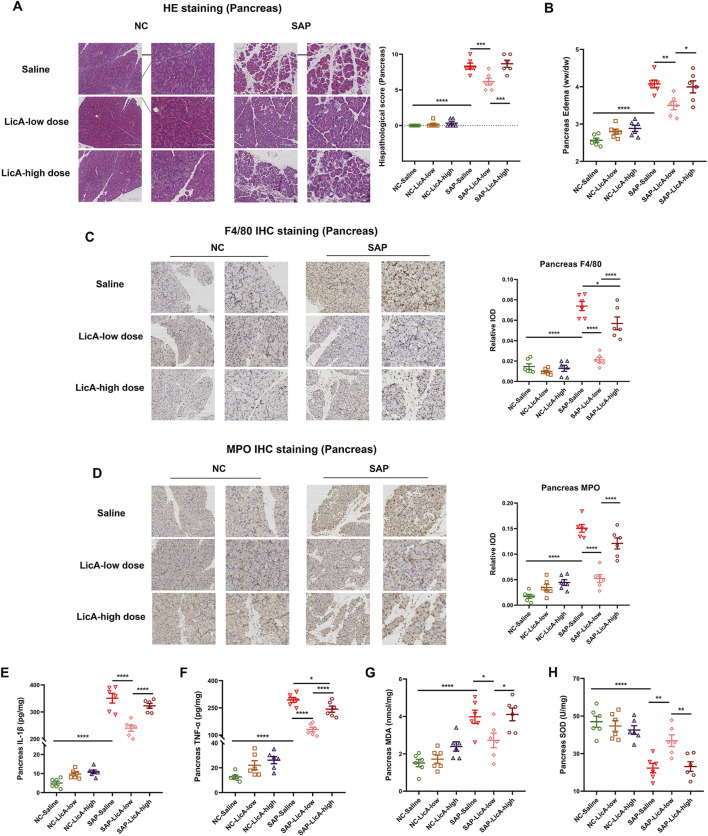
Differential pancreatic histopathological and inflammatory responses to low- and high-dose licochalcone A. SAP is induced by caerulein plus LPS and pancreatic tissues are collected at 24 h. **(A)** Representative H&E staining and histopathological scores showing severe acinar injury, edema, inflammatory infiltration, and necrosis in SAP mice. Low-dose LicA attenuates pancreatic damage, whereas high-dose LicA does not confer comparable pancreatic protection. **(B)** Pancreatic wet-to-dry weight ratios indicating tissue edema. Low-dose LicA reduces edema, while high-dose LicA shows no improvement. **(C,D)** Immunohistochemical staining for F4/80 and MPO demonstrating macrophage and neutrophil infiltration. Low-dose LicA decreases inflammatory cell infiltration, whereas high-dose treatment does not. **(E,F)** Pancreatic IL-1β and TNF-α levels. Low-dose LicA significantly reduces cytokine levels; high-dose LicA does not. **(G)** Pancreatic malondialdehyde (MDA) content. **(H)** Pancreatic superoxide dismutase (SOD) activity. SAP induction was associated with increased MDA and reduced SOD compared with NC. Data are shown as mean ± SEM (n = 6 per group). Scale bars: 200 μm (10×) and 100 μm (20×). Statistical comparisons are indicated as *p < 0.05, **p < 0.01, ***p < 0.001, ****p < 0.0001.

The combined administration of caerulein and lipopolysaccharide (LPS) reliably induced a SAP phenotype, marked by significant acinar cell swelling, cytoplasmic vacuolization, interstitial edema, inflammatory cell infiltration, and focal necrosis. Accordingly, pancreatic histopathological injury scores showed a marked and significant increase in the SAP group relative to controls (Figure 2A).

Pretreatment with LicA differentially affected SAP-associated pancreatic injury. Low-dose LicA (7.5 mg/kg) markedly attenuated pancreatic histological damage, as evidenced by reduced acinar cell injury and interstitial edema. The low-dose LicA treatment significantly reduced the pancreatic wet-to-dry weight ratio in SAP mice compared to the SAP group (Figures 2A,B). In contrast, high-dose LicA (15 mg/kg) did not alleviate pancreatic injury; histopathological injury scores and W/D ratios remained markedly elevated or were further increased compared with the SAP group (Figures 2A,B).

To assess inflammatory cell infiltration, immunohistochemical staining for F4/80 and MPO was performed. As shown in Figures 2C,D, SAP induction was associated with increased macrophage and neutrophil infiltration in pancreatic tissue. Low-dose LicA pretreatment significantly reduced F4/80^+^ macrophage and MPO^+^ neutrophil infiltration compared with the SAP group. In contrast, high-dose LicA treatment did not noticeably alter pancreatic F4/80 or MPO staining patterns relative to SAP mice (Figures 2C,D).

Local pancreatic inflammatory responses were further evaluated by measuring tissue levels of IL-1β and TNF-α. SAP mice exhibited significantly elevated pancreatic IL-1β and TNF-α levels. Low-dose LicA pretreatment significantly reduced the levels of both cytokines, whereas no significant improvement was observed in the high-dose LicA–treated SAP group (Figures 2E,F).

To further assess whether oxidative stress is associated with the dose-dependent pancreatic phenotype, pancreatic malondialdehyde (MDA) content and superoxide dismutase (SOD) activity were measured (Figures 2G,H). SAP induction was associated with increased MDA levels and reduced SOD activity compared with NC controls. In NC mice, high-dose LicA did not significantly change MDA content or SOD activity relative to NC-saline. In SAP mice, high-dose LicA did not further exacerbate MDA elevation or SOD reduction compared with SAP alone.

Taken together, these results indicate that low-dose LicA attenuates pancreatic histopathological injury and local inflammatory responses in SAP mice, whereas high-dose treatment did not to confer comparable pancreatic protection.

### 3.3 Intestinal barrier integrity and local inflammation following licochalcone A administration

**FIGURE 3:**
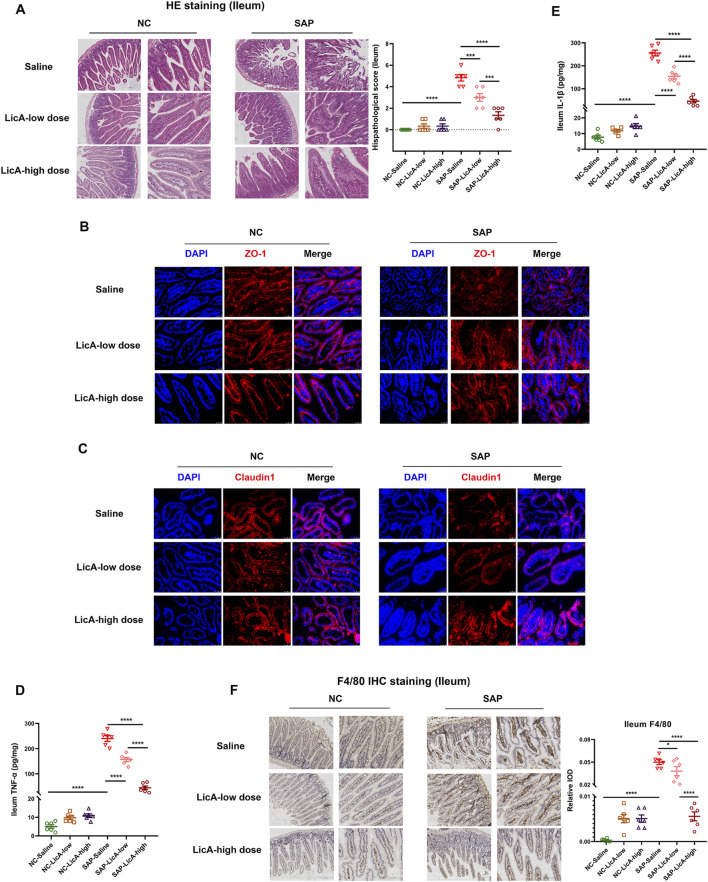
Licochalcone A preserves intestinal barrier structure and modulates ileal inflammation in SAP. Ileal tissues are analyzed 24 h after SAP induction. **(A)** Representative H&E staining showing mucosal disruption in SAP mice. Both low- and high-dose LicA improve intestinal morphology, with more pronounced structural restoration at the high dose. **(B,C)** Immunofluorescence staining of tight junction proteins ZO-1 and Claudin-1. SAP reduces and disrupts protein expression; LicA restores staining continuity, with stronger effects at the high dose. **(D,E)** Ileal IL-1β and TNF-α concentrations. SAP elevates IL-1β and TNF-α levels; LicA reduces them, with greater suppression at the high dose. **(F)** Immunohistochemical staining for F4/80 showing macrophage distribution. LicA treatment modulates F4/80^+^ cell patterns. Data are presented as mean ± SEM (n = 6 per group). Scale bars: 200 μm (10×) and 100 μm (20×) for H&E and IHC; 100 μm (20×) for immunofluorescence. Statistical significance is indicated as *p < 0.05, **p < 0.01, ***p < 0.001, ****p < 0.0001.

Given the critical role of intestinal injury in SAP pathogenesis, ileal tissues were examined to evaluate the impact of LicA on intestinal structure and the integrity of the barrier function. Representative H&E staining revealed apparent ileal injury in SAP mice, characterized by disruption of mucosal architecture and epithelial integrity (Figure 3A). Pretreatment with low-dose LicA alleviated SAP-associated ileal histopathological damage, while high-dose LicA treatment resulted in more pronounced improvement compared with the low-dose group (Figure 3A). To further evaluate intestinal barrier integrity, immunofluorescence staining targeting tight junction proteins was conducted.

SAP induction led to reduced and discontinuous expression of ZO-1 and Claudin-1 in the ileum (Figures 3B,C). Low-dose LicA pretreatment partially improved the continuity and expression of these tight junction proteins. Notably, high-dose LicA treatment resulted in more continuous ZO-1 and Claudin-1 staining patterns, consistent with improved intestinal barrier integrity.

Local intestinal inflammation was evaluated by measuring IL-1β and TNF-α levels in the ileum. Mice with SAP showed increased concentrations of both cytokines (Figures 3D,E). LicA pretreatment reduced the ileal levels of IL-1β and TNF-α, with the high-dose group exhibiting a greater suppression compared to the low-dose group.

Immunohistochemical staining of F4/80 demonstrated macrophage presence within the ileum (Figure 3F). LicA pretreatment modulated F4/80-positive cell distribution in the ileum, consistent with its effects on intestinal inflammation and barrier restoration.

Taken together, these findings indicate that both low- and high-dose LicA preserve intestinal structure and barrier-associated immune features in SAP. Notably, intestinal protection was observed at both doses, despite divergent systemic and pancreatic outcomes.

### 3.4 Microbial diversity and community restructuring under licochalcone A treatment

**FIGURE 4:**
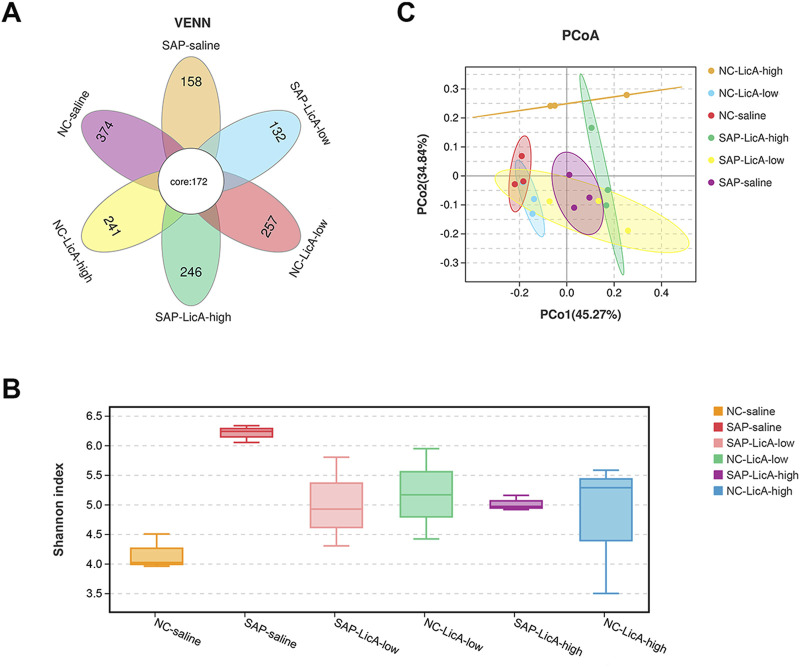
Dose-dependent remodeling of gut microbial diversity following licochalcone A treatment (exploratory analysis). Gut microbiota composition is analyzed by 16S rRNA sequencing of fecal samples. **(A)** Venn diagram showing shared and unique OTUs among groups, including 172 core OTUs and distinct OTU subsets associated with treatments. **(B)** Alpha diversity assessed by Shannon index. Shannon index was higher in SAP than in controls. Low-dose LicA was associated with a reduction toward control levels, whereas high-dose LicA remained similar to SAP. **(C)** Beta diversity assessed by principal coordinate analysis (PCoA). SAP separates from controls along PCo1 (45.27%). Low-dose LicA clusters closer to controls, while high-dose LicA occupies a distinct position along PCo2 (34.84%). Data are presented as mean ± SEM (n = 3 per group). Given the limited sample size, microbiota findings should be interpreted cautiously and are presented as exploratory/hypothesis-generating. Statistical procedures used for alpha- and beta-diversity comparisons are described in the Methods.

As an exploratory analysis, we assessed gut microbiota composition using 16S rRNA sequencing. A Venn diagram of operational taxonomic units (OTUs) revealed 172 core OTUs shared among all experimental groups, along with distinct OTU subsets specific to each treatment condition (Figure 4A). Notably, the high-dose LicA group exhibited a set of unique OTUs that differed from those observed in the low-dose LicA and SAP groups, indicating dose-associated differences in microbial community composition.

Alpha diversity analysis based on the Shannon index showed that SAP induction was associated with increased bacterial diversity compared to the control (P < 0.05). Low-dose LicA treatment reduced the Shannon index toward levels observed in NC mice, indicating a partial normalization of microbial community complexity. In contrast, high-dose LicA treatment did not restore baseline diversity and maintained a Shannon index comparable to that of the SAP group (Figure 4B).

Analysis of beta diversity via principal coordinate analysis (PCoA) further indicated dose-dependent shifts in microbial community structure. The SAP group was separated from the NC group along the primary coordinate (PCo1, 45.27%), while the low-dose LicA group clustered closer to NC, consistent with a partial recovery of microbiota composition (Figure 4C). In comparison, the high-dose LicA group occupied a distinct position in ordination space, showing separation from both NC and SAP groups along PCo2 (34.84%), showing that high-dose LicA was associated with a microbiota configuration that differed from both normal and SAP-associated patterns within the limits of this dataset.

Collectively, these exploratory analyses indicate that LicA treatment is associated with dose-dependent changes in the diversity and structural composition of the gut microbial community, with low-dose LicA tending toward partial normalization and high-dose LicA associated with a divergent microbial configuration.

### 3.5 Distinct microbial configuration associated with high-dose licochalcone A

**FIGURE 5:**
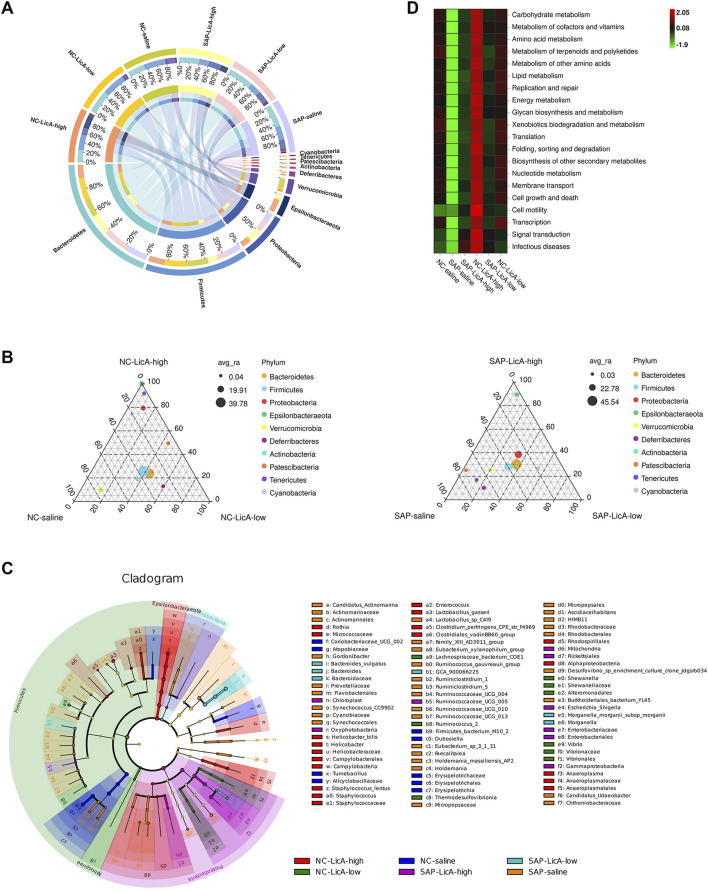
Exploratory taxonomic and functional profiling suggests distinct microbial features associated with high-dose licochalcone A exposure. Taxonomic and functional features of gut microbiota are evaluated using 16S rRNA sequencing–based analyses. **(A)** Chord diagram showing phylum-level composition. SAP decreases *Firmicutes* and increases *Bacteroidetes*. High-dose LicA is associated with increased relative abundance of *Epsilonbacteraeota*, including in NC mice. **(B)** Ternary plot showing OTU distributions across conditions. SAP-associated taxa cluster toward the SAP apex, whereas low-dose LicA groups show more balanced distributions. High-dose LicA in NC mice shows increased representation of multiple taxa toward the high-dose apex. **(C)** LEfSe analysis (LDA >2.0) identifying discriminative taxa. SAP enriches *Enterobacteriaceae* and *Vibrio*, while high-dose LicA shows increased host-derived mitochondrial signals. **(D)** PICRUSt2 functional prediction showing reduced representation of pathways related to infectious diseases and cell motility and increased pathways related to signal transduction and cofactor/vitamin metabolism in high-dose LicA groups. Data are presented as mean ± SEM (n = 3 per group). Statistical procedures and thresholds (e.g., LEfSe LDA cutoff) are described in the Methods. Given the limited sample size (n = 3 per group), LEfSe-identified taxa and PICRUSt2-based functional predictions should be regarded as preliminary/exploratory and hypothesis-generating rather than confirmatory; validation in larger cohorts is required.

To further characterize microbiota features associated with dose-dependent LicA treatment, phylum-level taxonomic distributions were examined using chord diagrams. In SAP–saline mice, the gut microbial community displayed a relative contraction of Firmicutes and an expansion of Bacteroidetes compared with NC controls (Figure 5A). Notably, the phylum Epsilonbacteraeota exhibited dose-dependent changes: SAP induction alone was associated with only a modest increase in Epsilonbacteraeota, whereas high-dose LicA treatment was accompanied by a marked expansion of this phylum. Moreover, the relative abundance of Epsilonbacteraeota in NC mice receiving high-dose LicA exceeded that observed in SAP mice treated with high-dose LicA, suggesting a potential association between high-dose LicA exposure and relative enrichment of this taxon within the limits of this small cohort independent of SAP induction.

Ternary plot analysis further suggested condition- and dose-associated taxonomic distributions (Figure 5B). OTUs assigned to Tenericutes, Deferribacteres, and Patescibacteria were preferentially distributed toward the SAP–saline apex, indicating their association with the SAP-related microbial configuration. In contrast, OTUs in both NC and SAP mice treated with low-dose LicA were more centrally distributed, consistent with a relatively balanced community structure. By comparison, high-dose LicA treatment in NC mice was associated with enrichment of multiple taxa, including Epsilonbacteraeota, Deferribacteres, Proteobacteria, Cyanobacteria, and Tenericutes, toward the NC–high-dose apex, reflecting a distinct microbiota profile associated with high-dose exposure.

Exploratory biomarker analysis using LEfSe (LDA score >2.0) identified group-associated taxa (Figure 5C). The SAP–saline group showed enrichment of taxa belonging to Enterobacteriaceae and *Vibrio*, whereas the high-dose LicA group exhibited an increased signal of host-derived mitochondrial sequences, which was observed predominantly in high-dose groups.

To further explore potential functional differences, PICRUSt2-based prediction was performed. High-dose LicA–treated groups displayed a functional profile distinct from NC and low-dose LicA groups, with reduced predicted activity in pathways related to “Infectious diseases” and “Cell motility” and increased representation of pathways involved in “Signal transduction” and “Metabolism of cofactors and vitamins” (Figure 5D).

Collectively, these exploratory analyses provide preliminary, hypothesis-generating signals suggesting that high-dose LicA is associated with a microbiota configuration different from both NC and SAP-associated states. Given the limited sample size (n = 3 per group), taxon-level and predicted functional inferences (LEfSe and PICRUSt2) should be interpreted cautiously and require validation in larger cohorts.

## 4 Discussion

In the present study, we observed a dose-dependent divergence between intestinal and pancreatic responses to LicA treatment in SAP. Low-dose LicA consistently attenuated pancreatic injury, reduced inflammatory cell infiltration, suppressed local and systemic inflammatory cytokine production, and partially normalized gut microbiota composition. In contrast, high-dose LicA, despite conferring pronounced protection of the intestinal barrier and inducing distinct alterations in gut microbial communities, did not alleviate pancreatic injury and was associated with aggravated acinar damage and persistent inflammation. These findings question the assumption that improvement of intestinal homeostasis uniformly translates into pancreatic protection in acute pancreatitis. These findings are consistent with the possibility that LicA exerts organ-specific effects within a limited dose range, although direct pharmacokinetic–toxicological profiling was not performed in the current study. Beyond this range, intestinal benefits may coexist with, rather than override, direct pancreatic vulnerability. High-dose LicA was associated with reinforced intestinal barrier integrity and a divergent microbial configuration, while pancreatic injury remained pronounced at this dose. Collectively, our results highlight a non-linear, organ-specific response to LicA treatment, underscoring the importance of dose optimization when targeting the gut–pancreas axis for therapeutic intervention.

This study notably identifies a disconnect between intestinal protection and systemic effects at elevated LicA doses. Both low- and high-dose LicA preserved ileal villus architecture, maintained tight junction protein expression (ZO-1 and Claudin-1), and attenuated mucosal inflammatory cell infiltration, indicating robust protection of the intestinal epithelial and immunological barrier. However, only low-dose LicA was accompanied by parallel improvements in systemic inflammatory cytokines and pancreatic injury, whereas high-dose LicA failed to show comparable pancreatic protection and was associated with exacerbated acinar damage and sustained systemic inflammation. These findings indicate that, in severe acute pancreatitis, preservation of intestinal structure and local immune quiescence, while important, may not be sufficient to ensure resolution of systemic inflammation or protection of distant organs (Garg and Singh, 2019). In parallel, SAP induction was associated with increased pancreatic MDA levels and reduced SOD activity compared with NC controls, consistent with prominent oxidative stress in pancreatic tissue. However, high-dose LicA did not further exacerbate these oxidative stress markers relative to SAP alone, suggesting that the aggravated pancreatic phenotype at the higher dose may involve mechanisms beyond additional oxidative stress.

Analysis of the gut microbiota provides additional context for this dose-dependent dissociation. Low-dose LicA partially normalized SAP-associated alterations in both α- and β-diversity, with microbial communities clustering closer to those of healthy controls (Zhang et al., 2021). In contrast, high-dose LicA did not restore baseline diversity but was associated with a distinct microbial configuration characterized by divergence in community structure and enrichment of specific taxa. Notably, expansion of Epsilonbacteraeota, predominantly *Helicobacter*, was observed in high-dose groups, including healthy mice treated with high-dose LicA, raising the possibility that some microbial shifts may reflect a direct drug-associated effect rather than a secondary consequence of SAP. At the same time, high-dose LicA was associated with increased representation of taxa commonly linked to short-chain fatty acid production, which may be consistent with the preserved intestinal morphology and barrier function observed at this dose (Patel et al., 2021). Together, these findings suggest that high-dose LicA induces a complex microbial remodeling that does not simply recapitulate either a healthy or SAP-associated state. An additional exploratory observation was the increased representation of host-derived mitochondrial sequence reads in high-dose groups. Mitochondrial DNA (mtDNA) is identified as a damage-associated molecular pattern that can trigger innate immune signaling pathways in experimental pancreatitis (Peng et al., 2024; Zhou et al., 2024). However, because 16S sequencing is not optimized for quantitative assessment of mitochondrial DNA and no direct validation assays were performed, this signal should be interpreted cautiously and regarded as hypothesis-generating. Whether mitochondrial-derived signals are biologically linked to the observed phenotype will require confirmation in larger cohorts and targeted mechanistic analyses. Given the limited group size (n = 3 per group), all taxon-level inferences from LEfSe and PICRUSt2 analyses should be regarded as exploratory. Accordingly, these microbiome analyses were not intended to provide definitive biomarker discovery or functional inference, but rather to generate hypotheses for validation in adequately powered cohorts.

A key question raised by our findings is whether the dose-dependent pancreatic outcomes of LicA are mediated through the gut–pancreas axis or reflect direct pancreas–drug interactions. The present study does not establish causality between microbiota alterations and pancreatic injury, and microbiota-related observations should therefore be interpreted as associative rather than causal. Future studies using antibiotic-mediated microbiota depletion, ideally combined with controlled recolonization and/or fecal microbiota transplantation, will be required to determine whether the divergent pancreatic outcomes are microbiota-dependent (Gheorghe et al., 2021). It should also be noted that antibiotic depletion can exert microbiota-independent effects on host immunity and barrier function and may influence pancreatitis severity in a model- and regimen-dependent manner; thus, complementary approaches are essential for causal inference (Soares et al., 2017). Therefore, the current data do not allow us to conclude that pancreatic effects are independent of gut modulation.

Taken together, our data are consistent with a framework in which LicA confers intestinal barrier protection across a range of doses, but only within a restricted pharmacological range does this local benefit translate into systemic and pancreatic protection. At higher doses, intestinal homeostasis may be preserved, yet direct pancreatic vulnerability and distinct microbiota remodeling coexist with unfavorable systemic outcomes. This non-linear, dose-dependent response is consistent with biphasic or hormetic patterns reported for other phytochemicals, in which adaptive or protective responses at lower concentrations give way to cellular or ecological stress at higher exposure levels (Liu M. et al., 2024). The doses of LicA (7.5 and 15 mg/kg) were selected based on prior *in vivo* studies demonstrating anti-inflammatory activity within a similar range (5–30 mg/kg), and were chosen as two representative points to test whether divergent phenotypic responses could be observed across a practical pharmacological range. Although pharmacokinetic measurements were not performed in the present study, these doses have been reported as biologically active without clear evidence of systemic toxicity in murine models (Kim et al., 2010; Zhang et al., 2021; Liu M. et al., 2024). Specifically, Zhang et al. showed that LicA doses up to 40 mg/kg effectively preserved intestinal integrity and modulated gut microbiota in a murine colitis model, further supporting that our highest dose of 15 mg/kg remains within a biologically active exposure range for the intestinal epithelium (Zhang et al., 2021). Nevertheless, the aggravated pancreatic phenotype observed at the higher dose in our SAP model raised the possibility that organ-specific sensitivity thresholds may exist under pathological conditions. Although nonspecific toxicity cannot be excluded in the absence of direct pharmacokinetic data, the concurrent intestinal protection observed at 15 mg/kg is consistent with the possibility of differential tissue susceptibility rather than uniform systemic toxicity. Future studies incorporating full dose–response curves and pharmacokinetic–toxicological analyses will be necessary to define these relationships more precisely.

This study has several limitations that should be acknowledged. First, pharmacokinetic parameters and tissue exposure levels of LicA were not measured, which limits interpretation of dose–response relationships and precludes distinction between organ-specific sensitivity and potential exposure-related effects. Second, microbiota analyses were associative and cross-sectional in nature and based on a limited sample size, and therefore taxonomic and functional inferences should be interpreted cautiously and regarded as exploratory. In particular, taxon-level biomarkers (LEfSe) and predicted functional profiles (PICRUSt2) should be considered hypothesis-generating and require validation in larger cohorts. Third, the findings derive from a single experimental SAP model and were evaluated at a single time point, which may not capture temporal or model-dependent variability. In addition, microbial communities were not experimentally manipulated, and mitochondrial-related observations were not directly validated. Accordingly, causality along the gut–pancreas axis cannot be established in the present study; future work using antibiotic-mediated microbiota depletion and/or fecal microbiota transplantation will be required to distinguish microbiota-dependent effects from direct pancreas–drug interactions. Finally, although oxidative stress markers were assessed in pancreatic tissue (MDA and SOD), apoptosis/necrosis-related readouts were not measured, limiting mechanistic attribution of the high-dose pancreatic phenotype. Future studies incorporating dose–exposure profiling, targeted microbial interventions, and direct mitochondrial assays will be required to further clarify the mechanisms underlying the dose-dependent divergence observed here.

Despite these limitations, the present study highlights the potential importance of dose optimization when targeting the gut–pancreas axis in severe acute pancreatitis. Our findings suggest that intestinal barrier protection alone does not guarantee systemic benefit and that higher dosing may be associated with a divergence between local intestinal effects and pancreatic or systemic outcomes (Du et al., 2024; Liu J. et al., 2024). These observations have implications for the translational evaluation of LicA and related compounds, highlighting the need to balance barrier-preserving properties against organ-specific tolerance and microbial remodeling.

## Data Availability

The raw data supporting the conclusions of this article will be made available by the authors, without undue reservation. 16S sequencing raw reads were deposited in the 502 NCBI Sequence Read Archive (Accession: PRJNA938304).
